# Revisited Globalization’s Impact on Total Environment: Evidence Based on Overall Environmental Performance Index

**DOI:** 10.3390/ijerph182111419

**Published:** 2021-10-29

**Authors:** Quan-Jing Wang, Yong Geng, Xi-Qiang Xia

**Affiliations:** 1 School of Business, Zhengzhou University, Zhengzhou 450001, China; wqj@zzu.edu.cn; 2 School of Environment and Science Engineering, Shanghai Jiaotong University, Minhang District, Shanghai 200240, China; ygeng@sjtu.edu.cn

**Keywords:** globalization, environmental performance, slowing globalization, GMM estimation

## Abstract

This study aims to examine the impact of globalization on environmental performance by employing panel data for 148 countries from 2001 to 2018, via the indicator of Environmental Performance Index to capture the overall environmental quality and KOF index to measure the multi dimensions of globalization. The empirical results suggest that globalization is critical to environmental performance, which is reliable while we conduct several robustness tests. Furthermore, if globalization increases, it would be beneficial for the environmental performance; moreover, among specific dimensions of globalization, economic globalization, social globalization and political globalization would bring about better environmental performance. Besides, the improvement of globalization, social globalization and political globalization would bring about better environmental performance, while that of economic globalization cannot change the overall environmental performance. Our study offers more insight into the relationship between globalization and environmental performance.

## 1. Introduction

Environmental pollution is one of the biggest economic and social challenges that humanity faces in the foreseeable future. Social problems, such as human diseases, extinctions of animals, and soil desertification which caused by environmental pollution have been generally gaining attention from scholars, governments and individuals. (Based on Our World in Data, air pollution contributed to 9% of deaths in 2017 globally, while the highest ratio is 15% in North Korea.) The Sustainable Development Goals of the United Nations are shaping the global political agenda, and countries around the world have made active commitments to the goals (United Nations, 2019), so, how to improve the environmental performance is critical to sustainable development. A large body of literature investigates the factor of environmental performance from the perspective of economic development, human resources, energy usage and environmental technologies both on the country and the firm level (Wen et al., 2016, Niu et al., 2017; Pickering et al., 2020; Yang et al., 2021) [1,2,3,4]. Furthermore, globalization is also a critical factor in environmental performance since the scale effect, composition effect and technology effect brought by it would change the economic activities, energy efficiency and technologies (Copeland and Taylor, 2013) [5]. 

Following this idea, some scholars tried to investigate the impact of globalization on the environment; however, there are still some areas that needed to be further investigated and the results are inconclusive (Zafar et al., 2019 [6]; Bilgili et al., 2020 [7]). Specifically, there are three opposing ideas about the influence of globalization on environmental performance. One is that globalization does harm to the environment (You and Lv, 2018; [8] Pata, 2021 [9]). Other studies suggest that globalization benefits environmental performance. Some scholars support this idea from the perspective of CO_2_ emissions (Ling et al., 2015; [10] Shahbaz et al., 2017 [11]). Some scholars stated that the impact of globalization on the environment varies among different countries (Hao, 2016; [12] Rudolph et al., 2017 [13]). Furthermore, there are also some problems among the existing empirical literature, such as only utilizing CO_2_ emissions to represent environmental damage (Figge et al., 2017; [14] Gill et al., 2018 [15]), or merely paying attention to a specific dimension of globalization such as trade openness or foreign direct investment (Shahbaz et al., 2019; [16] Zafar et al., 2019 [6]), as well as only employing data for few countries (Shahbaz et al., 2018; [17] Akadiri et al., 2019 [18]).

Reviewing the literature, we can find that, even though a large amount of it focuses on the relationship between globalization and the environment, the majority of studies hold that globalization has a negative effect on the environment, while a considerable amount of literature supports the contention that globalization benefits the environment; therefore, the conclusion is inconclusive, and this topic needs to be further examined. Based on the abovementioned analysis, we can conclude that there are some gaps among existing studies, for instance, limited work examined the impact of globalization on overall environmental performance, since most studies usually measure the environmental degradation by CO_2_ emissions. In addition, while most studies only focused on one specific dimension of globalization, such as trade or economic, limited work comprehensively investigated the role of globalization in environmental performance from the perspective of social, cultural and political globalization. Besides, limited work has attached importance to whether globalization’s change can affect the overall environmental performance. This scenario raises the following interesting questions that we aim to investigate: the first one is whether globalization influences overall environmental performance? If yes, can slowing or accelerating globalization affect national environmental performance? Furthermore, which dimension of globalization can significantly change the environmental performance? To the best of the authors’ knowledge, this paper is the first to empirically examine the impact of globalization on total environment, and the first to pay attention to the slowing or accelerating globalization.

To investigate such important issues, we collected multinational data covering 148 countries from 2001 to 2018 to conduct empirical testing by utilizing GMM estimation via the indicator of Environmental Performance Index and globalization variables provided by the KOF Swiss Institute (2020). The estimations support that globalization is critical to environmental performance. Hence, for the specific dimensions of globalization, economic globalization, social globalization and political globalization are beneficial for environmental performance. Finally, if the level of social globalization, political globalization or overall globalization experiences an increase, environmental performance would be also promoted, while slowing or accelerating economic globalization cannot change environmental performance. 

The main contributions of our study are as follows. Firstly, unlike previous studies’ focus on the globalization’s impact on CO_2_ emissions or ecological footprint, our study concentrates on the impact of globalization on total environment including air pollution, environmental health and ecosystem vitality, and investigates the role of globalization in overall environmental performance directly, which can offer more meaningful insight into environmental globalization and environmental politics, filling the gap among existing studies about the relationship between globalization and the environment (Shahbaz et al., 2016; [19] Bu et al.,2016; [20] Shahbaz et al., 2018; [17] Akadiri et al., 2019; [18] Karasoy and Akçay, 2019; [21] Khan and Ullah, 2019 [22]). Next, contrary to existing literature that examines the influence of globalization on the environment based on the data of few countries (Shahbaz et al., 2015; [23] Bilgili et al., 2020) [7], we carry out empirical tests by employing data for 148 multinational countries and GMM estimation, which can capture the dynamic progress of environmental performance and provide generally accepted conclusions worldwide. 

In addition, to examine how the specific dimension of globalization can influence environmental performance, we include the four variables of economic globalization, social globalization, culture globalization and political globalization to measure the multi dimensions of globalization, which can comprehensively examine the broader influence of globalization on environmental performance, unlike the existing literature, which only pays attention to trade openness (Ling et al., 2015; [10] Hakimi and Hamdi, 2016; [24]; Destek et al., 2018 [25]). Finally, we further pay our attention to the slowing or acceleration of globalization, to provide more detailed evidence on the influence of globalization on environmental performance under the era of anti-globalization, which is a novelty among previous studies.

The remaining parts of this paper are organized as follows. Section 2 reviews relevant literature and proposes the hypothesis. Section 3 offers detailed information on the variables, data and estimation. Section 4 provides the empirical results and the discussion. Section 5 concludes the main findings and offers the policy implications based on the conclusion.

## 2. Literature Review and Hypothesis 

### 2.1. Literature Review

With the growth of globalization over recent decades, vast studies have tried to cover the influence of globalization on the environment. A strand of studies proposed that globalization would increase CO_2_ emissions. For instance, Koçak and Şarkgüneşi (2018) [26] and Salahuddin et al. (2018) [27] empirically examined the influence of globalization on the environment via the indicator of FDI or trade openness and CO_2_ emissions (Karasoy and Akçay, 2019 [21]). Other studies hold that globalization would reduce the ecological footprint, such as Bilgili et al. (2020) [7], who empirically investigated the impact of globalization on environmental sustainability in Turkey during 1970–2014 by utilizing the ecological footprint as a proxy for environmental sustainability and KOF globalization measurements, whose results suggest that the improvement of financial globalization, politic globalization, trade globalization and interpersonal globalization would reduce the ecological footprint growth; see also Mrabet and Alsamara (2017) [28]. Le et al. (2016) [29] utilized particulate matter to measure the environment and empirically tested the impact of globalization on the environment, supporting that trade openness would improve particulate matter; a similar conclusion can be found in Wang et al. (2018) [30]. Some scholars pointed out that globalization would negatively affect the environment by utilizing the KOF index to measure globalization. For instance, Khan et al. (2019) [31] empirically examined the influence of globalization on CO_2_ emissions by utilizing data from Pakistan during 1971–2016 and suggested that the economic globalization, social globalization and political globalization exert a positive effect on CO_2_ emissions both in the short run and the long run. See also Salahodjaev (2016) [32].

Another idea holds that globalization is beneficial for environmental performance. For instance, Grainger (2005) [33] studied the role of globalization in environmental protection from the perspective of environmental globalization, and proposed that environmental globalization would bring about a globalizing response by NGOs and governmental or intergovernmental institutions to regulate environmental degradation. Similarly, Charfeddine (2017) [34] carried out an empirical investigation on the influence of trade openness on the ecological footprint by utilizing data for Qatar, whose results showed that trade openness would benefit the improvement of the ecological footprint; see also Figge et al. (2017) [14]. Some scholars also support globalization’s positive effect on the environment by utilizing the KOF index to measure globalization. For example, Zafar et al. (2019) [6] empirically examined the influence of globalization on CO_2_ emissions by utilizing data of OECD countries from 1990 to 2014 and pointed out that globalization would reduce national CO_2_ emissions. See also Akadiri et al. (2019) [18].

Moreover, some scholars stated that globalization’s impact on the environment varies among different dimensions. For instance, Rudolph et al. (2017) [13] examined the influence of globalization on the ecological footprint in 146 countries from 1981 to 2009, and found that specific dimensions of globalization exert different influences on the environment. Destek et al. (2018) [25] supported that economic globalization and social globalization would increase CO_2_ emissions while political globalization reduce the CO_2_ emissions. Besides, Haseeb et al. (2018) [35] and Salahuddin et al. (2019) [36] suggested that there exists no significant influence of globalization on CO_2_ emissions.

In addition to the inconclusive findings, while we review the empirical literature for the relationship between globalization and environmental performance, we find that there are some lacking which needed to be further investigated. The first problem of the empirical investigations for globalization’s impact on the environment is that a large body of studies utilized CO_2_ emissions or the ecological footprint to represent environmental damage (Bilgili et al., 2020 [7]; Winslow, et al. 2005 [37]; Kashwan, et al. 2017 [38]; Stern et al. [39]). Even CO_2_ emissions contribute to most of the environmental damage, it cannot capture the other specific aspects of environmental performance. The second problem of the empirical investigations of globalization’s impact on the environment is that most researchers only focused on one dimension of globalization such as trade openness or foreign direct invest (Hakimi and Hamdi, 2016; [24] Ling et al., 2015; [10] Destek et al., 2018 [25]), scant consideration is given to other dimensions of globalization such as social, cultural and political globalization (Ulucak et al., 2020; [40]). The third problem of the empirical investigations of globalization’s impact on the environment is that majority of the literature only empirically tests the influence of globalization on the environment by employing data of one country or few countries (Mrabet and Alsamara, 2017; [28] Charfeddine, 2017; [34] Shahbaz et al., 2018; [17] Salahuddin et al., 2018; [27] Akadiri et al., 2019 [18]). Empirical investigations based on few countries cannot offer more common implications for the role of globalization in the environment and may cause some unreliable results. 

### 2.2. Hypothesis

The influence of globalization on environmental performance can be understood as follows. Firstly, globalization may lead to the strengthening, expansion and deepening of global networks, which can bring higher global uniformity and connectivity of conventional environmental management (Grainger, 2005) [33]. Compared with countries that prefer protectionist policies, countries that participate in globalization are more likely to promote environmental protection through environmental globalization (Shahbaz et al., 2019 [16]). Furthermore, Kull et al. (2007) [41] supported the idea that globalization usually leads to the expansion and acceptance of neoliberal economic ideas, as well as support for the privatization and formal registration of land, implying that countries with higher levels of globalization would gain a better environmental performance by improving the utilization of land than countries with a lower level of globalization (Twerefou et al., 2017 [42]).

In addition, Meyfroidt and Lambin (2011) [43] suggested that, compared to countries carrying out protectionism measures, open countries can conduct better environmental management thorough the channels such as reforestation on abandoned land, international nongovernmental organizations on environments, multilateral environmental conventions, and aid agencies (Grau and Aide, 2008; [44] Hecht, 2010 [45]). Besides, they declared that with appropriate polices on forest regulations, globalization could yield benefits for the natural environment among open countries (Lambin and Meyfroidt, 2010) [46]. Finally, As suggested by Grossman and Krueger (1991) [47], globalization could help open countries to improve their environmental performance as well as to reduce the adverse effects on the environment by technology transfer from developed countries. Based on the abovementioned analyses, we propose following hypothesis:

**Hypothesis 1 (H1):**  *Globalization has a positive impact on overall national environmental performance as moderated by national economic, social and political openness.*

## 3. Variable, Data and Methodology

### 3.1. Variables 

Environmental Performance Index (*EPI*): In line with Yang et al. (2021) [4], we measure the environmental performance with the score of Environmental Performance Index (denoted by *EPI*), provided by the Yale Center for Environmental Law and Policy and the Center for International Earth Science Information Network at Columbia University. We select this variable for the following reasons: firstly, *EPI* is a comprehensive evaluation of overall national environmental quality, including most aspects of environmental impact such as air pollution, ecological footprint, forest, climate change, energy and so on (Solarin et al., 2017). [48] Secondly, aside from environmental health, *EPI* also reflects the facets of ecosystem vitality (Niu et al., 2017) [2]. Based on such advantages, this study prefers to measure environmental performance by utilizing *EPI*. A higher value of *EPI* represents better environmental performance.

Globalization (Global): Globalization indicators such as trade openness and inward foreign direct investment, as well as the data of the KOF Swiss Economic Institute are generally utilized in the existing literature (Feng et al., 2019; [49] Zheng et al., 2019 [50]), comparing to other indexes such as trade openness and IFDI that only capture the specific aspect of trade globalization, the KOF index can reflect overall globalization. The variable Globalization is a comprehensive index for globalization, which is a combination of three main dimensions of globalization—economic, social and political globalization, and so forth. To better capture the globalization of one country, we utilize the globalization data provided by the KOF Swiss Economic Institute (2020), denoted by Global. A higher value of Global represents a higher level of globalization. 

The main descriptions of *EPI* and the KOF index are listed in Table 1; we also provide the information about three issue categories, economic globalization (Global_economic), social globalization (Global_social) and political globalization (Global_political), which would be utilized to carry out robustness tests.

To control other factors of environmental performance, we include other economic, social and political variables into our estimations in accordance with previous studies (Galli et al., 2020; [51] Vanham et al., 2019; [52] Wang et al., 2019; [53] Wang et al., 2021 [54]).

(1) Per capita real GDP (GDP): Saboori et al. (2012) [55] proposed that there is a significant influence of economic development on GHG emissions in both the short term and the long term. More economic activities usually cause more GHG emissions or other pollutants, thus reducing environmental performance. To capture the effect of economic development on environmental performance, we incorporate it in the model, which is measured by per capita real GDP that is constant at 2010 US dollars (hereafter denoted by GDP).
(2) Proportion of manufacturing sectors to GDP (IND): As Romano (2013) [56] stated, GHG emissions from industrial sources account for 20% of total GHG emissions, with the cement, refinery, and iron and steel industries taking the highest shares. It is reasonable to infer that, while the share of manufacturing is higher, the carbon emissions experience an increase, which may lead to a worse environmental performance. Therefore, to control the influence of manufacturing on environmental performance, we set it as an explanatory variable, which is measured by the proportion of value added by the manufacturing sectors to GDP (denoted by Ind).
(3) Total population (POP): Nakicenovic et al. (2000) [57] studied the relationship between climate change and population, noting that the population is a major driving force of GHG emissions, implying that a greater population is more likely to cause a worse environmental performance. To test the potential influence of population on environmental performance, we include it as an explanatory variable, which is calculated by total population (hereafter denoted by POP).
(4) Population density (Density): Norman et al. (2006) [58] investigated the relationship between climate change and population density and concluded on a per capita basis that GHG emissions of low-density areas are 2–2.5 times more intensive than those in high-density areas. To control for the influence of population density on environmental performance, we include it in the model as an explanatory variable defined by people per square km (denoted by Density) (Wang et al., 2019) [53].
(5) Education (Education): A higher level of education usually means that citizens are more likely to produce or live in an environment friendly manner, as well as have an improved awareness of environmental protection. We thus include the level of education in our model, which is measured by the enrolment in secondary education according to Wang et al. (2019) [53], which is denoted by Education.
(6) Urbanization rate (Urban): Lin et al. (2017) [59] investigated the influence of population urbanization and land urbanization on environmental impact by employing data for Chinese cities, and concluded that urbanization is a key factor for environmental impact. We thus introduce urbanization in our model, which is measured by the share of urban residents to total population (denoted by Urban).
(7) Democracy (Democ): As Held and Hervey (2011) [60] noted, democracies have fewer restrictions on information, as scientists and concerned citizens have access to engage in events about climate change, and pressure from social institutions and individual citizens can push governments to take more measures to solve the problems caused by climate change and put more effort into protecting the environment. To control for the potential influence of democracy on environmental performance, we employ the indicator of Bjørnskov and Rode (2019) [61] (hereafter denoted by Democ).
(8) Utilization of land (Forest): National forests are beneficial for the mitigation of air pollution, as well as for the protection of soil and environmental health. We thus use the forest change to measure the utilization of land. Following previous literature (Meyfroidt and Lambin, 2011 [43]), we measure forest protection by the growth rate of forests, which is calculated by the percent of net forest change to forest area of the previous year, denoted by Forest.
(9) Environmental innovation (GI): The progress of environmental innovation is an effective way to improve energy efficiency for reducing energy consumption and mitigating GHG emissions (Grant et al., 2016; [62] Jorgenson et al., 2019 [63]). Shao et al. (2011) [64] captured green innovation by environmental innovation (denoted by GI), which highly relates to environmental protection R&D and the improvement of energy efficiency. Environmental innovation is measured by the total number of patents for environmental management, which is obtained from the Organization for Economic Co-operation and Development (OECD) Statistics. This variable is standardized based on the total population.

### 3.2. Data Source and Descriptive

Data for *EPI* are derived from the Yale Center for Environmental Law and Policy and the Center for International Earth Science Information Network at Columbia University, (Website of the Yale Center for Environmental Law & Policy: https://epi.envirocenter.yale.edu/epi-downloads. (accessed on 2 March 2021) Website of the Center for International Earth Science Information Network: https://sedac.ciesin.columbia.edu/data/collection/epi. (accessed on 21 February 2021) while data for Global are provided by the KOF Swiss Economic Institute. (https://kof.ethz.ch/en/forecasts-and-indicators/indicators/kof-globalisation-index.html (accessed on 22 February 2021) Data for Democ are obtained from Bjørnskov and Rode (2019) [61], while data for GI are derived from the Organization for Economic Co-operation and Development (OECD), and the data for the other variables are provided by the World Bank. We merge all data together based on the country and year; after deleting the missing values, we obtain an unbalanced panel data covering 148 countries from 2001 to 2018. All variables are taken into their natural logarithms by plus 1, except for Democ and Forest.

Table 2 provides the basic descriptive statistics of these variables. For *EPI*, the minimum, and maximum are 2.843, and 4.520, respectively; while the mean and standard deviation (S.D) are 4.110 and 0.312, respectively, suggesting that the environmental performance fluctuates less among such sample countries. Next, we pay attention to globalization: the min, mean, median and max of Global are 3.282, 4.133, 4.160 and 4.522, respectively, while the S.D is 0.256. For other variables, the mean and median of GDP are 8.663 and 8.704, with an S.D of 1.486, suggesting that the economic performance varies among these countries. 

### 3.3. Estimating Methods—GMM

As suggested by previous studies, panel estimation is more valid than time series and cross-section estimation, since it includes the two dimensions of time and individual which can improve the efficiency and offer more information about individuals’ dynamic progress, as well as solving the potential problems caused by missing variables (Wen et al., 2016; [1] Wang et al., 2021 [54]). 

In line with Wen et al. (2016) [1], we also conduct empirical testing for the impact of globalization on environmental performance using system GMM estimation, which can control the lag term of *EPI*, meaning to include the dynamic progress of *EPI*, which is given below:

(1) $$EPI_{it}=\alpha_{1}EPI_{i,t-1}+\beta_{1}Global_{it}+\beta ’X+u_{i}+u_{t}+\varepsilon_{it},$$

where i=1,2,3…N  is the dimension of individuals and t=1,2,3…T is the time dimension. EPI is the environmental performance, while $EPI_{i,t-1}$ is the first lag of it, to test the dynamic progress of environmental performance; Global is the variable of globalization, and the other terms are similar to those in Equation (1). X represents the control variables, β stands for the corresponding coefficient, $u_{i}$ and $u_{t}$ capture the individual and time fixed effects, respectively; i=1,2,3…N  stand for the individual country; t=1,2,3…T refers to the year; and $\varepsilon_{it}$ is the error term.

## 4. Empirical Results

### 4.1. Baseline Results

Table 3 presents the results of system GMM estimation for globalization’s influence on environmental performance. We only consider Global and the year fixed effect in column (1), and include other factors which may affect environmental performance in the remaining regressions. It can be found that that the coefficient of Global in column (1) is 0.222, passing the significance test at the 1% level with a positive symbol, suggesting that a higher level of globalization benefits environmental performance. Furthermore, while we include the economic performance and industrial structure into the model, the coefficient of Global in column (2) is 0.269, which is significantly positive at the 1% level, again confirming the positive influence of globalization on environmental performance. Similarly, while we take other factors, such as population, education, IFDI, urbanization, trade openness and democracy, into the estimation, the results in column (3)–(6) all support globalization’s positive impact on environmental performance. In addition, while we pay attention to the lag term of *EPI*, we can obtain that all coefficients of L. *EPI* in columns (1)–(6) pass the significance test at the 1% level with a positive symbol, indicating that environmental performance is a dynamic process; an earlier effort on environmental protection would produce better outcomes for the current environmental performance. The main reason for this phenomenon is that higher globalization usually brings about the technology effect and general concerns about environmental protection, which eventually result in a better environmental performance (Copeland and Taylor, 2013) [5].

Our baseline results offer strong evidence that globalization tends to be better for environmental performance, which is similar to the research of Akadiri et al. (2019) [18] who argued that environmental globalization does some good for environmental protection, as well as Zafar et al. (2019) [6], who proposed that globalization would bring about the displacement of national deforestation.

To guarantee the credibility of our earlier finding, we further conduct several robustness tests such as changing the estimations, changing the measurement of globalization and constructing the new samples.

### 4.2. DIFF-GMM Estimation

First, we re-estimate the impact of globalization on environmental performance by employing the difference GMM estimation. Table 4 presents the results of difference GMM estimation for globalization’s influence on environmental performance. Similar to Table 3, we add the other control variables successively in columns (1)–(6). It can be found that that the coefficient of *Global* in column (1) is 1.021, which is significantly positive at the 1% level. Furthermore, while we include other variables in the estimation, the results in columns (2)–(6) all support globalization’s positive impact on environmental performance. The results in Table 4 are similar to those in Table 3, suggesting that our results are reliable.

### 4.3. Change the Measurement of Globalization

Secondly, Rudel (2002) [65] argued that globalization means not only economic globalization, but is also a multifaceted phenomenon with essential cultural and political dimensions (see also Grainger (2005) [33] as well as Khan and Ullah (2019) [22]). There are three main issue categories in the KOF index—economic globalization, social globalization and political globalization. For economic globalization, unlike earlier international trade and IFDI which cause serious environmental adverse effects, “environmentally friendly” is an important characteristic of current trade activities, meaning that the scale effect brought by economic globalization is weaker (Zafar et al., 2019) [6]. Meanwhile, the technique effect brought about by economic globalization would do some good to change the manner of producing activities or the application of energy-saving technologies, which may improve environmental performance (Ling et al., 2015) [10]. For social globalization, higher interpersonal or informational globalization means that the communication between domestic citizens and foreigners is more convenient, which offers individuals more access to information about environmental protection, influencing citizens with the global concern about the environment, thus putting more pressure on governments to protect the environment (You and Lv, 2018) [8]. Additionally, social globalization would lead to a greater transfer of knowledge or technologies between a host country and other countries, as well as the human capital, thus offering the country more power to achieve a better environmental performance. For political globalization, a higher level of political globalization usually brings about more international organizations and treaties; with the growing importance of environmental protection worldwide, more international NGOs and treaties often push governments to conduct measures to improve the national environmental performance (Kull et al., 2007) [41].

We thus measure the specific dimensions of globalization, such as economic globalization, social globalization and political globalization (More detailed information of such variables can be seen in the KOF Swiss Index.) The results for these variables are listed in columns (1)–(3) of Table 5. The coefficient of Global_Economic is 0.289, which is significant and positive at the 1% level, indicating that a higher level of economic globalization usually brings about better environmental performance. Similarly, the results in columns (2) and (3) show that the coefficients of Global_Social, and Global_Political are 0.526, and 0.083, respectively; both are significant at the 1% level, suggesting that social globalization and political globalization would also positively affect environmental performance. These results are in line with Kull et al. (2007) [41], who supported the idea that globalization is a multifaceted phenomenon and political globalization affects the environment, and Khan and Ullah (2019) [22], who argued that economic globalization and social globalization would affect CO_2_ emissions.

### 4.4. Slowing or Accelerating Globalization 

With the slowdown of globalization, trade protectionism is on the rise; both OECD countries and non-OECD countries have tried to bring their manufacturing sectors back to their home countries (Zhu and Jiang, 2019) [66]. Considering this phenomenon, we further query whether the slowing or acceleration of globalization affects environmental performance by setting a new variable, which is calculated by the difference of Global, Global_economic, Global_Social, Global_Political, whose results are listed in Table 6. It can be seen that the coefficient of ΔGlobal in column (1) is 0.647, which is significantly positive at the 1% level, suggesting that the increase of globalization would bring about a better environmental performance. Similarly, while we pay attention to the specific dimensions of globalization, the results of which are given in columns (2)–(4), we can obtain that the improvement of social globalization and political globalization also promotes environmental performance, while the improvement of economic globalization would not change the environmental performance. 

### 4.5. New Samples

Similar to Wang et al. (2019) [53], we re-conduct the empirical test based on new samples by removing the outliers which possess the first and last 10% of *EPI*, the results of which are given in Table 7. We can find that the coefficient of Global, Global_Economic, Global_Social and Global_political is significantly positive at the 1% level. The results in Table 7 support our earlier statements.

## 5. Conclusions

In the context of environmental politics, this paper focuses on the impact of globalization on environmental performance by utilizing multinational data for 148 countries during the period 2001–2018, along with system GMM estimation via the indicators of globalization, given by the KOF Swiss Economic Institute (2020), and the environmental performance index (*EPI*), which is a comprehensive evaluation of overall national environmental quality covering environmental health, water, air pollution, biodiversity and habitat, forests, fisheries, agriculture, climate change, and energy. The estimation supports the hypothesis that globalization exerts a significantly positive influence on environmental performance, meaning that higher levels of globalization would bring about better environmental performance. This finding is credible as we carry out several robustness tests by employing another estimation of difference, GMM estimation, or considering specific dimensions of globalization such as economic globalization, social globalization, and political globalization, as well as setting new samples by removing the outliers. Furthermore, for the specific dimensions, economic globalization, social globalization and political globalization would improve the environmental performance. Finally, we also investigated whether the improvement of globalization can affect environmental performance, suggesting that with increasing social globalization, political globalization and overall globalization, the environmental performance would also experience an increase. 

Our findings offer several policy implications for policy-makers. First, given that globalization is critical to environmental protection, governments can take advantage of globalization to spread the idea of environmental protection, namely environmental globalization, and can improve the structure of economic activities or international trade led by economic globalization. Secondly, among the multi-dimensions of globalization besides economic globalization, social globalization benefits environmental performance; the governments should increase efforts to stimulate the communication between domestic societies and abroad, which may increase citizens’ cognition of environmental protection against the background that environmental protection and sustainable ecosystems are generally accepted globally. Furthermore, since the increase of globalization would do some good to gain a better environmental performance, against the background of anti-globalization and post-novel coronavirus, those governments preferring better environmental quality should take measures to control the coronavirus and spread the importance of globalization for environmental protection, which can improve the global environment totally. In addition, since the international trade or FDI may bring some environmental adverse effects caused by the displaced environmental pollution from developed countries. While governments participate in globalization, they should pay attention to those trade activities or FDI that may cause environmental damage. Governments can levy an extra tax on these products or services to spend on environmental protection. Similarly, governments can require the importers of products that have environmental adverse effects to pay carbon fees based on the carbon emissions generated during the production process of the product, as the European Union has proposed. Finally, it is worth noting that our study may have limitations, since the KOF index we utilized in this study assigns equal weight to economic globalization, social globalization and political globalization while calculating the total level of globalization; however, countries may have different weights in terms of these dimensions according to their economic or social development. This essential problem should be further investigated once we have obtained a more accurate database for the weight of different countries. 

## Figures and Tables

**Table 1:** Main description for variables of *EPI* and Global.

Variables	Sub-Indices	Index
*EPI*	Environmental health (40%)	Including air quality, sanitation & drinking, water, heavy metals and waste management
	Ecosystem vitality (60%)	Including biodiversity & habitat, ecosystem services, fisheries, climate change, pollution emissions, agriculture and water resources
Global	Including economic globalization, social globalization and political globalization, weight for each one is 33%	
Global_economic (33%)	Trade globalization (50%)	Including trade in goods and services, as well as trade partner diversification
	Financial globalization (50%)	Including FDI, Portfolio investment, international debt, reserves and income payments
Global_social (33%)	Interpersonal globalization (33%)	Including transfers, migration, international voice traffic and tourism
	Informational globalization (33%)	Including patent applications, international students and high technology exports
	Cultural globalization (33%)	Including trade in cultural goods and personal services, trademark applications, McDonald’s restaurant and IKEA stores
Global_political (33%)		Including embassies, UN peace keeping missions, and International NGOs

**Table 2:** Data Descriptive.

Variable	N	Mean	S. D	Min	Median	Max
*EPI*	1956	4.110	0.312	2.843	4.205	4.520
Global	1956	4.133	0.256	3.282	4.160	4.522
GDP	1956	8.663	1.486	5.277	8.704	11.626
IND	1956	3.232	0.373	1.067	3.249	4.486
POP	1956	15.965	1.908	9.880	16.097	21.025
Density	1947	4.230	1.224	0.945	4.330	7.607
Education	1956	4.326	0.484	2.021	4.502	5.106
Urban	1956	3.985	0.469	2.270	4.129	4.615
Democ	1939	0.698	0.459	0.000	1.000	1.000
Forest	1940	0.025	0.938	−6.227	0.000	8.838
GI	1945	0.398	1.869	0.000	0.004	17.864

**Table 3:** The impact of globalization on *EPI*—SYS-GMM estimation.

	(1)	(2)	(3)	(4)	(5)	(6)
L.EPI	0.794 ***	0.671 ***	0.668 ***	0.631 ***	0.519 ***	0.477 ***
	(70.578)	(51.360)	(46.755)	(38.118)	(30.529)	(23.715)
Global	0.222 ***	0.269 ***	0.287 ***	0.381 ***	0.529 ***	0.465 ***
	(16.200)	(15.787)	(14.551)	(15.690)	(14.776)	(11.607)
GDP		0.001	−0.002	−0.008 **	−0.009 *	−0.013 **
		(0.252)	(−0.683)	(−2.440)	(−1.813)	(−2.013)
IND		0.048 ***	0.046 ***	0.040 ***	0.049 ***	0.061 ***
		(10.319)	(9.886)	(7.802)	(7.544)	(7.018)
POP			−0.006	−0.002	0.043 ***	0.062 ***
			(−1.418)	(−0.379)	(6.769)	(6.920)
Density			−0.005 ***	−0.013 ***	−0.028 ***	−0.032 ***
			(−3.838)	(−6.214)	(−7.858)	(−7.357)
Education			−0.006 **	−0.006 **	0.002	0.001
			(−2.104)	(−2.056)	(0.587)	(0.161)
Urban					−0.006	0.015
					(−0.459)	(1.016)
Democ						0.048 ***
						(5.348)
Forest						0.005
						(1.591)
GI						0.006 *
						(1.850)
Year FE	yes	yes	yes	yes	yes	yes
Cons	−0.052 ***	0.055	0.341 ***	0.311 ***	0.579 ***	0.926 ***
	(−3.077)	(1.334)	(7.526)	(5.523)	(8.304)	(11.389)
N	1956	1956	1947	1947	1883	1848
AR (1)	−6.351	−6.293	−6.227	−6.264	−5.933	−5.868
AR (1)-P	0.000	0.000	0.000	0.000	0.000	0.000
AR (2)	−1.180	−1.136	−0.695	−0.775	−1.331	−1.278
AR (2)-P	0.238	0.256	0.487	0.439	0.183	0.201
Hansen-P	0.428	0.505	0.468	0.505	0.745	0.645

Notes: ***, **, and * indicate statistical significance at the 1%, 5%, and 10% levels, respectively. Z-statistics are in parenthesis.

**Table 4:** Robustness test—DIFF-GMM estimations.

	(1)	(2)	(3)	(4)	(5)	(6)
L.EPI	0.314 ***	0.344 ***	0.304 ***	0.304 ***	0.216 ***	0.209 ***
	(12.049)	(13.514)	(12.008)	(11.652)	(8.826)	(6.048)
Global	1.021 ***	0.874 ***	0.724 ***	0.723 ***	0.856 ***	1.111 ***
	(9.476)	(6.725)	(6.289)	(5.687)	(5.107)	(4.887)
GDP		−0.056 **	−0.012	−0.008	−0.020	−0.030
		(−2.233)	(−0.569)	(−0.380)	(−0.716)	(−0.856)
IND		0.058 ***	0.018	0.023	−0.012	−0.028
		(3.533)	(1.221)	(1.571)	(−0.585)	(−1.288)
POP			−0.012 *	−0.012 *	0.009	0.004
			(−1.711)	(−1.695)	(0.933)	(0.270)
Density			−0.012	−0.183	−5.181 **	−2.422
			(−0.016)	(−0.222)	(−2.181)	(−1.068)
Education			0.243	0.431	5.629 **	2.769
			(0.305)	(0.497)	(2.270)	(1.175)
Urban					0.039	0.090
					(0.404)	(0.816)
Democ						0.100 ***
						(3.461)
Forest						−0.009 *
						(−1.829)
GI						0.020
						(0.869)
Year FE	yes	yes	yes	yes	yes	yes
N	1704	1704	1695	1695	1640	1609
AR (1)	−5.211	−5.556	−5.433	−5.280	−4.097	−3.723
AR (1)-P	0.000	0.000	0.000	0.000	0.000	0.000
AR (2)	−0.972	−0.951	−0.957	−0.975	−1.397	−0.651
AR (2)-P	0.331	0.342	0.338	0.330	0.162	0.515
Hansen-P	0.351	0.165	0.261	0.323	0.699	0.855

Notes: ***, **, and * indicate statistical significance at the 1%, 5%, and 10% levels, respectively. Z-statistics are in parenthesis.

**Table 5:** Robustness test—specific dimension of globalization.

	(1)	(2)	(3)
L.EPI	0.579 ***	0.444 ***	0.568 ***
	(27.648)	(18.999)	(26.041)
Global_Economic	0.289 ***		
	(10.536)		
Global_Social		0.526 ***	
		(15.019)	
Global_Political			0.083 ***
			(4.567)
GDP	0.000	−0.032 ***	0.014 ***
	(0.051)	(−4.394)	(2.641)
IND	0.072 ***	0.023 **	0.052 ***
	(6.114)	(2.382)	(5.928)
POP	−0.012 ***	−0.002	−0.022 ***
	(−2.690)	(−0.570)	(−5.029)
Density	0.004	−0.011 **	0.006 *
	(1.079)	(−2.364)	(1.719)
Education	0.062 ***	−0.028 **	0.099 ***
	(7.887)	(−2.185)	(10.121)
Urban	−0.010	0.053 ***	0.004
	(−0.561)	(3.116)	(0.232)
Democ	0.065 ***	0.047 ***	0.080 ***
	(7.014)	(4.837)	(11.096)
Forest	−0.005 **	0.007 **	0.001
	(−2.424)	(2.401)	(0.401)
GI	0.008	0.005	0.013 **
	(1.464)	(0.952)	(2.254)
Year FE	yes	yes	yes
Cons	0.786 ***	0.651 ***	1.128 ***
	(6.769)	(6.332)	(10.773)
N	1848	1848	1848
AR (1)	−5.964	−5.754	−6.264
AR (1)-P	0.000	0.000	0.000
AR (2)	−1.347	−1.219	−0.717
AR (2)-P	0.178	0.223	0.473
Hansen-P	0.658	0.704	0.705

Notes: ***, **, and * indicate statistical significance at the 1%, 5%, and 10% levels, respectively. Z-statistics are in parenthesis.

**Table 6:** Robustness test—slowing or accelerating globalization.

	(1)	(2)	(3)	(4)
L.EPI	0.644 ***	0.616 ***	0.626 ***	0.616 ***
	(25.481)	(26.309)	(24.854)	(27.532)
ΔGlobal	0.647 ***			
	(7.426)			
ΔGlobal_Economic		−0.020		
		(−0.788)		
ΔGlobal_Social			0.435 ***	
			(8.183)	
ΔGlobal_Political				0.162 ***
				(5.722)
GDP	0.018 ***	0.020 ***	0.021 ***	0.016 ***
	(3.009)	(3.540)	(3.664)	(2.803)
IND	0.044 ***	0.054 ***	0.055 ***	0.053 ***
	(4.439)	(5.688)	(5.566)	(6.028)
POP	−0.014 ***	−0.012 ***	−0.010 ***	−0.014 ***
	(−3.343)	(−3.593)	(−2.598)	(−4.523)
Density	0.006	0.006 *	0.004	0.005
	(1.506)	(1.703)	(1.139)	(1.522)
Education	0.078 ***	0.079 ***	0.087 ***	0.086 ***
	(7.681)	(8.836)	(9.197)	(9.368)
Urban	0.012	0.004	−0.001	0.015
	(0.708)	(0.220)	(−0.083)	(0.931)
Democ	0.071 ***	0.087 ***	0.088 ***	0.086 ***
	(6.635)	(10.222)	(9.502)	(10.808)
Forest	−0.002	−0.001	−0.001	−0.004 *
	(−0.719)	(−0.483)	(−0.458)	(−1.687)
GI	0.016 *	0.013 *	0.010	0.017 **
	(1.760)	(1.658)	(1.229)	(2.201)
Year FE	yes	yes	yes	yes
Cons	1.127 ***	1.134 ***	1.065 ***	1.151 ***
	(9.140)	(10.170)	(9.476)	(11.649)
N	1848	1848	1848	1848
AR (1)	−6.300	−6.336	−6.362	−6.302
AR (1)-P	0.000	0.000	0.000	0.000
AR (2)	−0.549	−0.632	−0.005	−0.483
AR (2)-P	0.583	0.527	0.996	0.629
Hansen-P	0.678	0.599	0.788	0.646

Notes: ***, **, and * indicate statistical significance at the 1%, 5%, and 10% levels, respectively. Z-statistics are in parenthesis.

**Table 7:** Robustness test—Middle 80% sub-sample.

	(1)	(2)	(3)	(5)
L.EPI	0.702 ***	0.747 ***	0.667 ***	0.774 ***
	(29.461)	(30.346)	(30.671)	(39.933)
Global	0.277 ***			
	(9.560)			
Global_Economic		0.097 ***		
		(5.708)		
Global_Social			0.314 ***	
			(9.652)	
Global_Political				0.019 **
				(2.071)
GDP	−0.011 ***	−0.004	−0.027 ***	−0.007 *
	(−2.893)	(−0.922)	(−4.224)	(−1.758)
IND	0.012 *	0.011 *	0.002	0.009
	(1.867)	(1.804)	(0.329)	(1.302)
POP	−0.019 ***	−0.008 **	−0.004	−0.012 ***
	(−6.848)	(−2.511)	(−1.461)	(−3.852)
Density	−0.009 ***	−0.005 **	−0.015 ***	−0.002
	(−3.099)	(−1.988)	(−4.097)	(−0.964)
Education	0.025 ***	0.042 ***	−0.012	0.050 ***
	(3.390)	(6.610)	(−0.918)	(7.257)
Urban	0.020	0.027 *	0.051 ***	0.045 ***
	(1.428)	(1.953)	(3.900)	(3.484)
Democ	0.020 ***	0.030 ***	0.018 **	0.032 ***
	(3.341)	(4.744)	(2.493)	(5.443)
Forest	−0.001	−0.004 ***	0.001	−0.002 *
	(−0.448)	(−3.013)	(0.555)	(−1.692)
GI	0.019 ***	0.016 ***	0.026 ***	0.022 ***
	(4.255)	(2.689)	(4.648)	(3.198)
Year FE	yes	yes	yes	yes
Cons	0.391 ***	0.557 ***	0.402 ***	0.651 ***
	(4.568)	(5.712)	(3.251)	(6.815)
N	1365	1365	1365	1365
AR (1)	−5.455	−5.404	−5.347	−5.502
AR (1)-P	0.000	0.000	0.000	0.000
AR (2)	0.200	0.177	0.522	0.581
AR (2)-P	0.841	0.860	0.602	0.561
Hansen-P	0.864	0.925	0.527	0.869

Notes: ***, **, and * indicate statistical significance at the 1%, 5%, and 10% levels, respectively. Z-statistics are in parenthesis.

## Data Availability

The data for globalization is obtained from KOF Swiss Economic Institute: https://kof.ethz.ch/en/forecasts-and-indicators/indicators/kof-globalisation-index.html, the data for environmental performance is derived from Yale Center for Environmental Law and Policy (https://epi.yale.edu/) and Center for International Earth Science Information Network at Columbia University (https://sedac.ciesin.columbia.edu/data/collection/epi), data for green innovation is obtained from OECD statistics (https://stats.oecd.org/), data for other variables can be found at World Development Indicator (https://databank.worldbank.org/reports.aspx?source=wdi-database-archives-(beta) (accessed on 27 October 2021).
